# Laparoscopic triple-ureteral ureteroureterostomy in a patient with ureteral triplication: A case report

**DOI:** 10.1097/MD.0000000000031580

**Published:** 2022-11-04

**Authors:** Saisai Liu, Rugang Lu, Yunfei Guo, Gao Xiucheng

**Affiliations:** a From the Division of Pediatric Urology, Children’s Hospital of Nanjing Medical University, Nanjing, Jiangsu Province, China; b From the Department of Radiology, Children’s Hospital of Nanjing Medical University, Nanjing, Jiangsu Province, China.

**Keywords:** case report, congenital abnormality, laparoscopic triple-ureteral ureteroureterostomy, ureteral triplication, vesicoureteral reflux

## Abstract

Ureteral triplication (UT) is a very rare disorder, with a challenging diagnosis and varied therapeutic methods. In the past, the treatment usually involved heminephrureterectomy of the stunted moiety. Here, we reported a case of complete UT that was treated by laparoscopic triple-ureteral ureteroureterostomy (UU).

**Patient concerns:**

A 2-year-old girl presented with frequent urine incontinence. Intravenous pyelography and voiding cystourethrography revealed a 3-segment left kidney with pelvi-ureteric dilation of the upper and middle moieties and grade IV to V vesicoureteral reflux. Laparoscopic triple-UU was successfully performed in the child, after which vesicoureteral reflux and urinary incontinence disappeared completely, hydronephrosis was improved, and hemirenal resection was avoided.

**Diagnosis:**

Based on the imageological examination results, the final diagnosis of the 2-year-old girl was as follows: left 3 renal pelvis with complete ureteral duplication, combined with upper and middle hydronephrosis, and upper and middle vesicoureteral reflux grade IV to V.

**Intervention:**

Laparoscopic triple-ureteral ureteroureterostomy was performed under general anesthesia.

**Outcomes:**

The patient recovered smoothly without complications after surgery. At 6 months follow-up, ultrasonography of the urinary system showed that hydronephrosis of the dilated kidney segment was attenuated.

**Lessons:**

Laparoscopic triple-ureteral UU was successful in our patient. For children with duplex kidney and multiple ureteral duplications, minimally invasive urinary tract reconstruction can be a suitable intervention.

## 1. Introduction

Ureteral triplication (UT) is a rare disorder of the upper urinary tract. Wrany described the first triple kidney repeat malformation in 1870,^[1,2]^ after which fewer than 100 cases of UT have been reported.^[3]^ Surgical intervention typically involves removal of duplicated renal segment and ureters with poor function. Vesicoureteral reimplantation or ureteral anastomosis has also been performed in a few cases when the duplicated renal segment function was sufficient. After surgical intervention, patients often experience poor function of duplicated renal segment dysplasia. Herein, we reported a case of complete UT that was treated by laparoscopic triple-ureteral ureteroureterostomy (UU). This is the first report on laparoscopic triple-ureteral UU in a patient with complete UT, which can be a method of urinary tract reconstruction for the treatment of UT.

## 2. Case presentation

**Figure 1.**
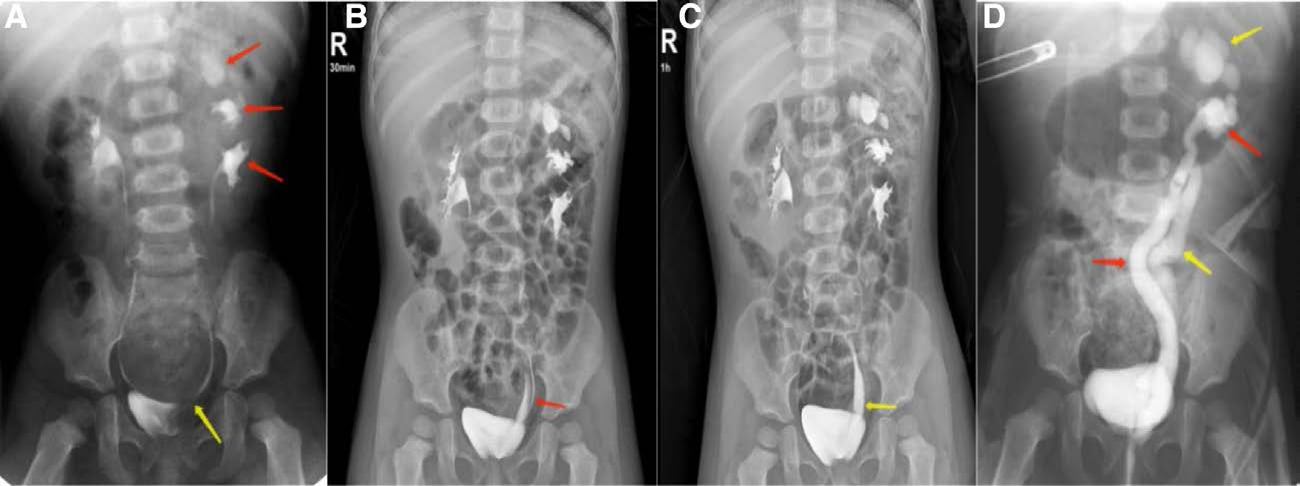
Intravenous pyelography and VCUG examination of the patient. (a) IVP images showed the 3 renal pelves on the left side (red arrows), the upper pelvis was significantly dilated, the upper and middle renal calyx were round and blunt, and the ureter connected to the lower renal pelvis was normally opened into the bladder (yellow arrow). (b) The dilated end of the middle ureter (red arrow) at 30 min after contrast agent injection. (c) The dilated end of the upper ureter (yellow arrow) at 1 h after contrast agent injection. (d) The VCUG image showed dilated upper (yellow arrow), middle renal pelves (red arrow) and associated ureters, dilated left upper and middle pelves, obtuse renal calyx, and grade IV–V vesicoureteral reflux. IVP = intravenous pyelography, VCUG = voiding cystourethrography.

A 2-year-old girl was admitted to the division of pediatric urology at the Children’s Hospital of Nanjing Medical University on October 18, 2020, with the chief complaint of urine incontinence. She had a history of 2 febrile urinary tract infections (the specific condition was unknown) and frequent urine incontinence for more than 1 year. There was no abnormality on general physical examination, and her biochemical and urine routine indexes were normal. Urinary ultrasound examination and magnetic resonance urography showed 3 renal pelves on the left side. IVP examination (at 15 minutes, 30 minutes, and 1 hour, respectively) clearly showed the presence of the left 3 groups of renal pelves: the upper group was expanded with spherical changes; the middle group was dilated with blunt renal calyx, while the renal calyx morphology of the lower group was normal. The renal pelves of the 3 groups were connected to the ureters, wherein the upper and middle ureters were dilated (Fig. 1a and b) and the lower group of ureter was normal (Fig. 1c). Voiding cystourethrography showed 2 dilated ureteral opacities on the left (grade IV–V), reflux of the left upper and middle ureters, dilated upper and middle pelves, and blunting of the renal cup (Fig. 1d). Static renal scan with the isotope technetium 99 m-dimercaptosuccinic acid demonstrated that the left kidney accounted for 55% of the total kidney function. The upper, middle and lower pole kidney segment accounted for 12%, 19%, and 69% of the total left kidney function, respectively.

Based on the above examination results, the final diagnosis of the child was as follows: left 3 renal pelvis with complete ureteral duplication, combined with upper and middle hydronephrosis, and upper and middle vesicoureteral reflux grade IV to V. The child underwent surgery on October 30, 2020.

**Figure 2.**
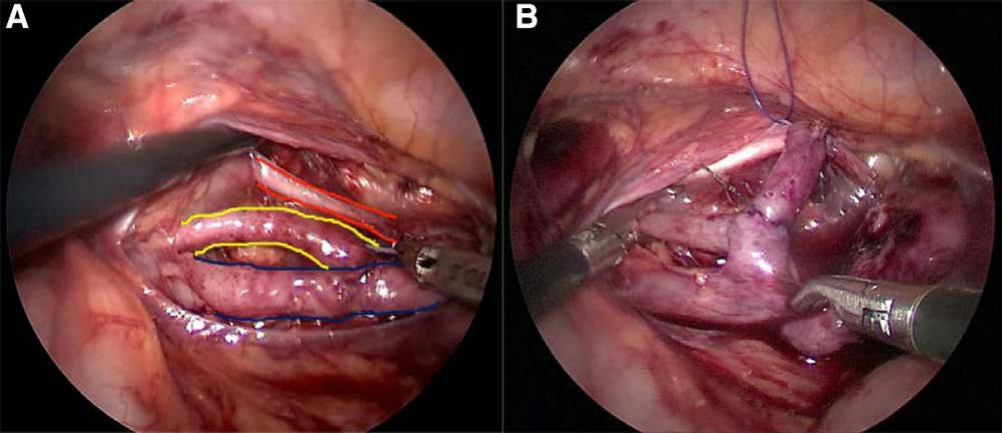
(a) The 3 ureters on the left side, the upper ureter (blue double line) and the middle ureter (yellow double line) were dilated, while the lower ureter (red double line) was normal. (b) The upper, middle, and lower ureters after 2 end-sided anastomoses.

**Figure 3.**
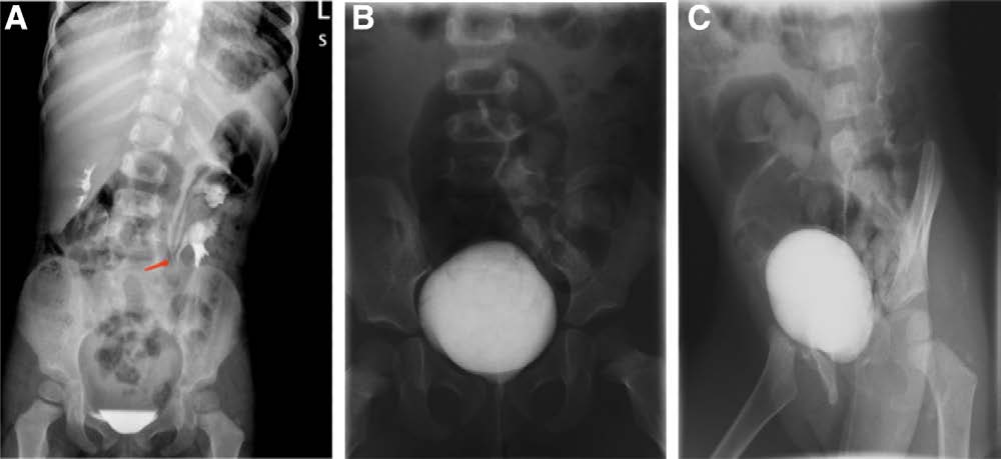
The IVP image taken at 6 months post-operation showed that the 3 left ureters converged at the lower edge of the L4 vertebral body (arrow). VCUG images at 6 months post-operation showed normal bladder morphology (b) orthostatic tablet, (c) left anterior oblique tablet, no vesicoureteral reflux. IVP = intravenous pyelography, VCUG = voiding cystourethrography.

During the operation, retrograde pyelography confirmed the previous diagnosis. After opening the posterior peritoneum in the pelvic segment, we found that the upper and middle ureters shared a sheath. We carefully separated the left 3 ureters, and found that upper and middle ureteral tube diameters were approximately 1 cm dilated, while the lower ureter had normal diameter (Fig. 2a). First, UU was performed between the upper and middle ureters. Next, an F4 120 mm DJ tube was inserted down the distal end of the ureter along the cannula needle at approximately 1.5 cm of the longitudinal split of the lateral margin of the lower ureter. The second operation of the middle and lower ureters (UU) was performed at the distal end of the upper and middle ureteral anastomosis (Fig. 3). The 3 ureters were anastomosed by UU twice and finally formed a stepped structure (Fig. 2b). There was no significant leakage from the anastomotic opening. The proximal upper and middle ureters were removed after the distal ureters were clamped by homelock clamps. The operation duration was 75 minutes. At 26 days post-operation (dpo), the DJ tube was removed, and the child was discharged from the hospital on November 24, 2020.

**Figure 4.**
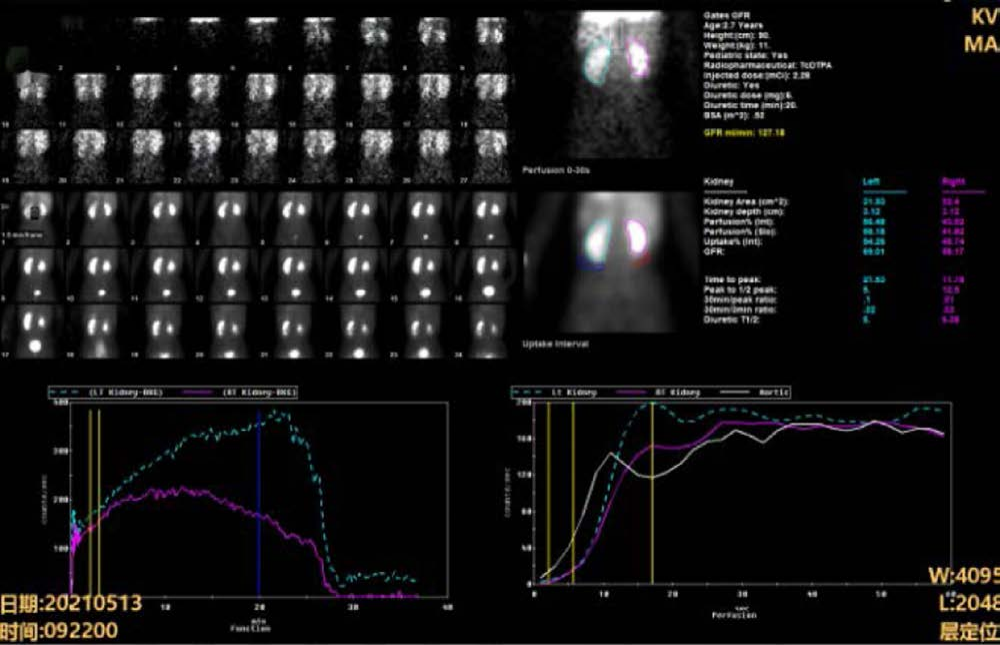
A postoperative interventional GFR scan showed that the GFR of the left kidney was 69.01 mL/min and that of the right kidney was 58.17 mL/min. There was no obstruction of flow in either ureter. GFR = glomerular filtration rate.

**Figure 5.**
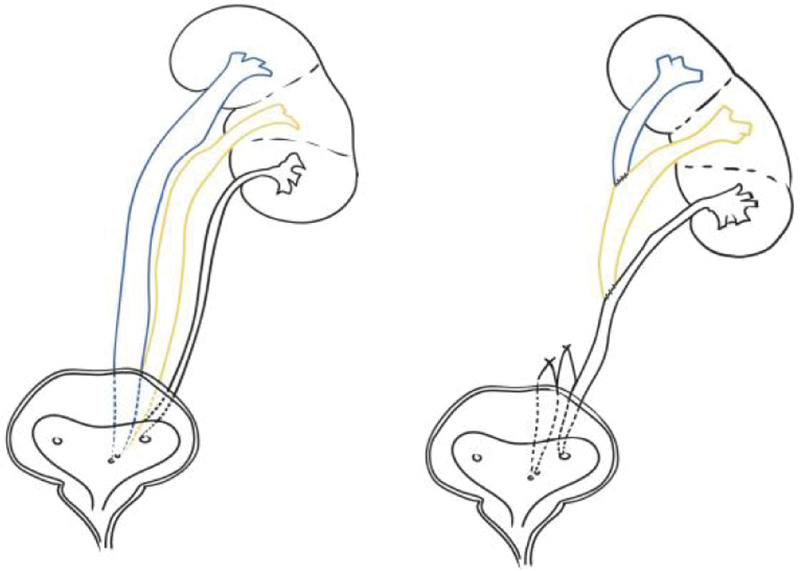
The anatomical situation before and after surgery.

A follow-up was performed at 6 months after surgery on May 18, 2021. Ultrasonography of the urinary system showed that hydronephrosis of the dilated kidney segment was attenuated. IVP clearly showed that the upper, middle and lower ureters on the left side converged at the lower edge of the L4 vertebral body, and the contrast agent was excreted normally (Fig. 4a). The upper and middle vesicoureteral reflux had completely disappeared (Fig. 4b and c). A postoperative ^99m^Tc-DTPA furosemide renogram suggested a glomerular filtration rate of 69.01 mL/min for the left kidney and 58.17 mL/min for the right kidney (Fig. 5), with no signs of ureteral obstruction. The size and morphology of the kidneys, and filtration function were normal. The patient is currently under close follow-up 18 months after surgery and is in good health.

## 3. Discussion and conclusions

The mechanism of UT is similar to that of duplicated renal occurrence, but the former is relatively rarer. The clinical symptoms of UT include recurrent urinary tract infections, perineal dampness, urinary incontinence, enuresis, or pain due to ectopic ureteral bulge.^[4,5]^ Some cases are asymptomatic, which are detected incidentally during other tests. The diagnosis requires comprehensive examination by ultrasonography, magnetic resonance urography, IVP, retrograde pyelography and renal function scan. Smith^[2]^ summarized all previously documented cases in 1946, classifying UT into 4 types. According to his classification criteria, our case was classified as the first type, which was a completely independent one-sided repetitive malformation of 3 ureters, each with its own independent renal collection system, and 3 distal ureters independently opening into the bladder.

Given the major variations in anatomy and renal function, comprehensive perioperative imaging data and accurate assessment of renal function are necessary to guide surgical management. Most of the surgical intervention methods reported in children with UT involve resection of the dysplastic renal segment and ureter.^[6]^ Cases of upper urinary tract reconstruction surgery are rarely reported. Youngson reported a 6-year-old girl with type II triplication and an ectopic ureter that drained a well-functioning upper renal segment and was preserved by ipsilateral UU, with excellent postoperative recovery.^[7]^ Ashwin Shekarp reported a 14-year-old girl who was diagnosed with left Smith type IV triple ureteral duplication with left upper kidney grade IV vesicoureteral reflux. The child underwent left low UU surgery and developed transient urinary leakage post-operation, which disappeared after comprehensive management.^[8]^ Irfan Wahyudi reported a 10-month-old female infant who was diagnosed with bilateral UT (right Smith type II, left Smith type III), accompanied by left vesicoureteral grade V reflux. The child underwent left laparoscopic Lich-Gregoir extravesical ureteral reimplantation. The urinary leakage and UTIs disappeared after the operation.^[9]^ Unlike previous cases, in our case, the child was severely refluxed with the upper and middle bladder ureters on the left side, and the lower ureters were normally opened in the bladder triangle with abnormal drainage. To reduce the burden and further trauma to the bladder, we did not perform ureteral bladder reimplantation. The preoperative renal function scan of the child suggested that the left upper and middle renal segments were functioning. In order to retain the left renal unit as much as possible and also minimize the risk of damage to the kidney, we performed 3 ureteral end-side anastomosis and drained the upper and middle renal pelves of the hydronephrosis through urinary tract reconstruction. UU surgery was first proposed by Foley in 1928.^[10]^ With the development of laparoscopic techniques, laparoscopic UU has been successfully applied for the treatment of children with duplex kidneys, due to its significant advantages.^[11]^ There are no relevant case reports on the repeated collection system of 3-ureteral end-side anastomosis treatment. This is the first case report of successful anastomosis of 3 ureters with UU twice, which expands the scope of UU surgery.

Each patient with UT requires an individualized management and treatment strategy. Herein, we implemented a special surgical procedure--laparoscopic triple-ureteral UU to preserve the hydronephrotic renal segment for the first time, and the follow-up results showed that the operation was successful. We recommend UU reconstruction of multiple ureters to treat multiple ureteral duplication, and the repeated kidney segment should not be removed unless necessary.

## Acknowledgments

This research did not receive any specific grant from funding agencies in the public, commercial, or not-for-profit sectors.

## Author contributions

**Visualization:** Saisai Liu, Yunfei Guo, Gao Xiucheng.

**Writing – original draft:** Saisai Liu.

**Writing – review & editing:** Saisai Liu, Rugang Lu.
